# Expression and Characterization of Alkaline Phosphatase from *Cobetia amphilecti* KMM 296 in Transiently Transformed Tobacco Leaves and Transgenic Calli

**DOI:** 10.3390/plants13243570

**Published:** 2024-12-21

**Authors:** Peter Adeolu Adedibu, Yulia Aleksandrovna Noskova, Yulia Anatolievna Yugay, Daria Mikhailovna Ovsiannikova, Elena Anatolievna Vasyutkina, Olesya Dmitrievna Kudinova, Valeria Petrovna Grigorchuk, Yury Nikolaevich Shkryl, Liudmila Aleksandrovna Tekutyeva, Larissa Anatolievna Balabanova

**Affiliations:** 1 School of Advanced Engineering Studies, Institute of Biotechnology, Bioengineering and Food Systems, FEFU, 10 Ajax Bay, 690922 Vladivostok, Russialbalabanova1@gmail.com (L.A.B.); 2 Laboratory of Marine Biochemistry, G.B. Elyakov Pacific Institute of Bioorganic Chemistry, Far Eastern Branch, Russian Academy of Sciences, Prospect 100-letya Vladivostoka 152, 690022 Vladivostok, Russia; 3 Federal Scientific Center of the East Asia Terrestrial Biodiversity, Far Eastern Branch, Russian Academy of Sciences, 159 Stoletija Str., 690022 Vladivostok, Russiayn80@mail.ru (Y.N.S.)

**Keywords:** bacterial alkaline phosphatase, marine bacteria, PhoA family, tobacco, recombinant protein, plant molecular farming

## Abstract

Alkaline phosphatase (ALP) of the PhoA family is an important enzyme in mammals, microalgae, and certain marine bacteria. It plays a crucial role in the dephosphorylation of lipopolysaccharides (LPS) and nucleotides, which overstimulate cell signaling pathways and cause tissue inflammation in animals and humans. Insufficient ALP activity and expression levels have been linked to various disorders. This study aims to produce recombinant ALP from the marine bacterium *Cobetia amphilecti* KMM 296 (CmAP) in transformed leaves and calli of *Nicotiana tabacum* and to elucidate the influence of the plant host on its physical and chemical properties. *N. tabacum* has proven to be versatile and is extensively used as a heterologous host in molecular farming. The *alp* gene encoding for CmAP was cloned into the binary vectors pEff and pHREAC and transformed into *N. tabacum* leaves through agroinfiltration and the leaf disc method for callus induction using *Agrobacterium tumefaciens* strain EHA105. Transformed plants were screened for recombinant CmAP (rCmAP) production by its enzymatic activity and protein electrophoresis, corresponding to 55 kDa of mature CmAP. A higher rCmAP activity (14.6 U/mg) was detected in a homogenate of leaves bearing the pEFF-CmAP construct, which was further purified 150-fold using metal affinity, followed by anion exchange chromatography. Enzymatic activity and stability were assessed at different temperatures (15–75 °C) and exposure times (≤1 h), with different buffers, pHs, divalent metal ions, and salt concentrations. The results show that rCmAP is relatively thermostable, retaining its activity at 15–45 °C for up to 1 h. Its activity is highest in Tris HCl (pH 9.0–11.0) at 35 °C for 40 min. rCmAP shows higher salt-tolerance and divalent metal-dependence than obtained in *Escherichia coli*. This can be further explored for cost-effective and massively scalable production of LPS-free CmAP for possible biomedical and agricultural applications.

## 1. Introduction

*Cobetia amphilecti* KMM 296, a marine bacterium, has recently garnered increasing scientific interest [1,2,3] owing to its rich diversity of important enzymes [4,5,6,7], with potential applications in biotechnology, medicine, and agriculture. Among these enzymes is alkaline phosphatase (ALP), which plays an important role in several biological processes, including phosphate metabolism and neutralizing excess signaling extracellular LPS and nucleotides [4]. This enzyme is noted for its high catalytic activity and stability over a wide pH range [8], making it suitable for various industrial processes, bioremediation, and other applications.

Microbial alkaline phosphatases (ALPs) are noted to play a significant role in the adaptation of bacteria to diverse environments [4], especially when competing with bacteria and fungi; they also facilitate biofilm growth and degradation [9], bioremediation, and mineralization of host invertebrate exoskeletons [10]. Alkaline phosphatase from *C. amphilecti* KMM 296 (CmAP), along with alkaline phosphatase from the human resident symbiont *E. coli*, has received research attention lately. These enzymes belong to the same protein structural family, PhoA, as human alkaline phosphatases, which include intestinal alkaline phosphatase (IAP), tissue-nonspecific alkaline phosphatase (TNAP), and placental alkaline phosphatase (PLAP). PhoA refers to the alkaline phosphatase protein family, characterized by shared structural and enzymatic properties, including a broad substrate specificity and key roles in phosphate metabolism. CmAP and *E. coli* ALP exhibit considerable similarity, suggesting a common evolutionary ancestry and a potential anti-inflammatory role in multicellular host organisms [4,11]. Intestinal alkaline phosphatase (IAP) plays a pivotal role in the detoxification of LPS and the regulation of the inflammatory response; a deficiency of this enzyme has been linked to an increased incidence of LPS-induced inflammatory conditions [11]. It is established that IAP activity declines with age, resulting in a corresponding rise in LPS levels [12,13] and associated health disorders; thus, exogenous ALP supplementation is a highly effective strategy for maintaining optimal health [4,14]. There is a growing interest in CmAP as a potential enzyme drug with an IAP-like function, suitable for supplementation to detoxify LPS [4,11]. Several studies have been performed to establish heterologous expression systems for producing adequate-quality human ALP due to its requirement for specific glycosylation. Only a few trials have proceeded to the clinical stage [14,15]. The *E. coli* PhoA enzyme is notably ineffective without artificial mutations [4]. Conversely, recombinant production of the highly active CmAP was successfully carried out in *E. coli* cells [8,16]. Nevertheless, the absence of LPS in the host expression system is essential to obtain recombinant CmAP without LPS contamination; the plant system adequately fills the gap and should be explored.

Plant molecular farming is a rapidly advancing field in biotechnology, allowing the cost-effective production of functional recombinant proteins of therapeutic and industrial importance in sufficient amounts using plant systems [17,18]. It has been demonstrated that tobacco species, such as *N. tabacum* and *Nicotiana benthamiana,* are highly effective heterologous expression systems for recombinant enzymes of interest with a wide range of applications [19,20]. These species are often referred to as the “white mice” of plant science due to their genetic modification sensitivity and widespread usage as a platform for early research in recombinant protein production [21]. Recombinant proteins are primarily expressed in the leaves rather than the flowers, reducing the risk of inadvertent gene leakage to the environment via pollens or seeds [20]. *Nicotiana* species are well suited for effective genetic transformation and produce high enzymatic yields from their large leaf biomass [20]. Furthermore, tobacco plants are free from human endotoxins and other contaminants, such as LPS, which could limit the therapeutic application of recombinant ALP produced in bacterial expression systems. This is advantageous when expressing recombinant enzymes of therapeutic importance in tobacco plants.

This study explores the heterologous expression of alkaline phosphatase from the PhoA structural family, native to the marine bacterium *C. amphilecti*, in *N. tabacum*. This study aims to compare the recombinant enzyme yield in tobacco callus and leaf tissues using different vectors, genetic constructs, and methods. The physical and chemical properties of the recombinant enzyme rCmAP, isolated from the transformed tobacco, have been characterized. This holds the potential for producing a high-purity, LPS-free enzyme that could be considered for therapeutic use in veterinary and human medicine.

## 2. Results

### 2.1. Genetic Constructs and Molecular Characterization of Recombinant Plant-Based Bacterial Alkaline Phosphatase CmAP

**Figure 1:**
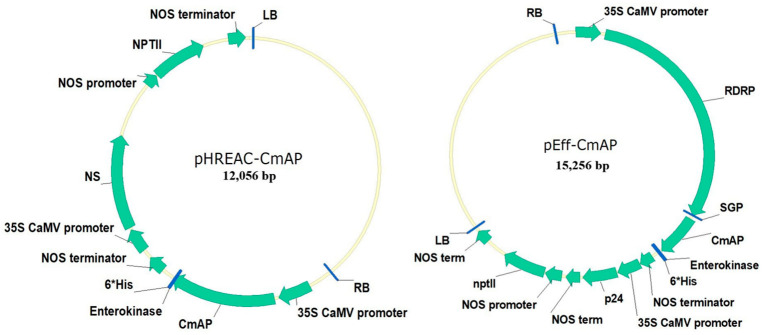
Schematic representation of the genetic constructs expressing the *C. amphilecti* KMM 296 alp gene encoding rCmAP in *N. tabacum*. The constructs feature the 35S CaMV promoter driving CmAP expression. Key elements include the NOS promoter and terminator for regulatory control, NPTII as a selectable marker, and left (LB) and right (RB) border regions for *Agrobacterium*-mediated plant transformation. Additional components include viral suppressors of RNA silencing (p24 and NS), a subgenomic promoter region (SGP), and an RNA-dependent RNA polymerase (RDRP) for RNA replication. Both constructs include a 6×His tag and an enterokinase cleavage site for simplified purification.

The recombinant vectors pHREAC-CmAP and pEff-CmAP (Figure 1) were used to transiently transform *N. tabacum* leaves and generate transgenic calli expressing the alp gene, which is responsible for the synthesis of the recombinant *C. amphilecti* KMM 296 protein CmAP (rCmAP). The vectors pHREAC and pEff are known to facilitate the high expression of recombinant enzymes [22,23].

**Figure 2:**
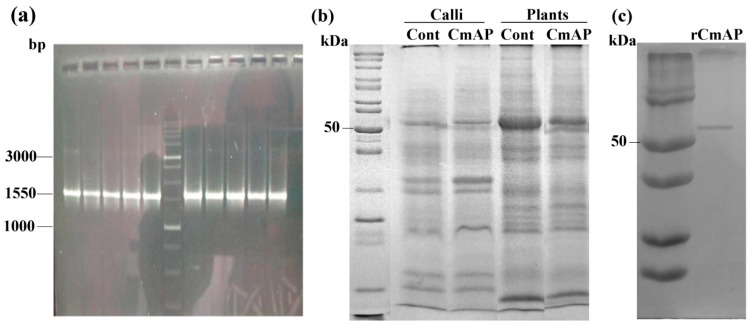
(**a**) Agarose gel electrophoresis showing amplification of the full-length *alp* gene for rCmAP (1550 bp) in transformed *N. tabacum* calli. Lane M: DNA base pair (bp) ladder used as a size standard. (**b**) SDS-PAGE analysis of crude protein extracts from control (Cont) and transformed (CmAP) calli and leaves of *N. tabacum*. (**c**) Purified recombinant CmAP (rCmAP) from transgenic plants after Ni-agarose affinity purification. Lane M: molecular weight (MW) marker (New England Biolabs, MA, USA).

The presence of the *alp* gene, encoding for rCmAP, was confirmed in the transgenic *N. tabacum* plant by polymerase chain reaction (PCR), with a 1550 bp band visualized on 1.2% agarose gel electrophoresis (Figure 2a).

The purified rCmAP, as determined by SDS-PAGE analysis, had a molecular weight of 55 kDa, corresponding to that of the native enzyme [7,8]. Production rates of 0.115 μg per gram of fresh leaf weight were achieved, with this protein accounting for approximately 5.2% of the total soluble protein. The resulting gel images are presented in Figure 2b,c.

### 2.2. Recombinant Gene Expression for rCmAP Production

**Figure 3:**
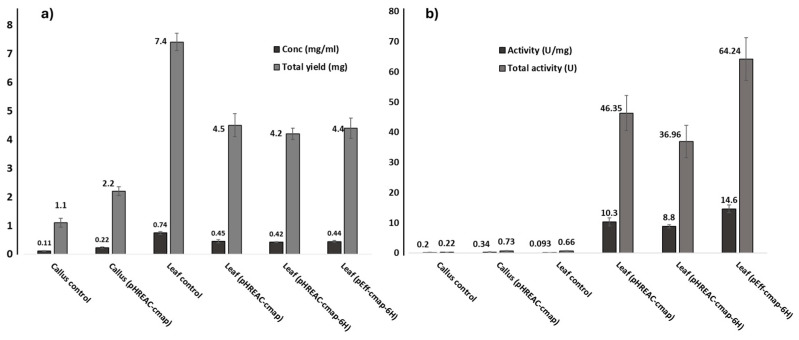
Recombinant CmAP yield and activity in transgenic *N. tabacum* leaves and calli lysates. (**a**) Shows the protein concentration and yield, and (**b**) shows the enzymatic activity of rCmAP. Corresponding values of each metric are presented on the *y*-axis. Data are presented as mean ± standard error.

Recombinant CmAP was successfully expressed in the leaves and calli of *N. tabacum*, with a considerable yield and enzymatic activity. The total yield of the active recombinant enzyme rCmAP was 2.2–7.4 mg, obtained from a 10-mL homogenate of 2 g transformed tissues. The analysis results showed a specific activity of 0.093–14.6 U/mg among the assessed samples. In general, the activity and yield of the recombinant enzyme rCmAP were higher in the transformed leaf tissues compared to the calli. The average specific activity was 11.2 U/mg and 0.34 U/mg, while the total activity was 49.2 U and 0.73 U in the leaves and calli tissues, respectively (Figure 3).

The pEff-CmAP vectors exhibited the highest rCmAP enzymatic activity and yield in transformed leaves (specific activity—14.6 U/mg, total activity—64.2 U), higher than that observed for pHREAC-CmAP constructs (specific activity—8.8–10.3 U/mg, total activity—4.5–36.96 U) (Figure 3). The recombinant CmAP exhibited a twofold lower expression level in callus compared to *N. tabacum* leaves (Figure 3).

The homogenate derived from the *N. tabacum* leaves carrying the pEff-CmAP-6H vector yielded the recombinant enzyme with the highest specific activity (14.6 U/mg). The selected sample was subjected to further purification to facilitate the characterization of the recombinant enzyme rCmAP (Table 1).

A total of 0.227 mg of the recombinant protein rCmAP was obtained from the initial 2 g of the transformed leaves. A higher specific activity was recorded at each purification step, from the initial 14.6 U/mg from the homogenate solution to 733.3 U/mg at the final concentration step using Mono-Q, indicating a 50-fold purification level of rCmAP (Table 1). Compared to *E. coli*-expressed rCmAP at each purification step, the enzymatic yield and activity of the rCmAP expressed in *N. tabacum* are lower. The final purification step yields a specific activity of 4052.6 U/mg for *E. coli*-expressed rCmAP, compared to 733.30 U/mg for *N. tabacum*-expressed rCmAP. The rCmAP obtained from *N. tabacum* constituted 5.2% of the total protein content, higher than the 0.37% yield in *E. coli*.

### 2.3. Effect of Temperature and pH on rCmAP Activity

**Figure 4:**
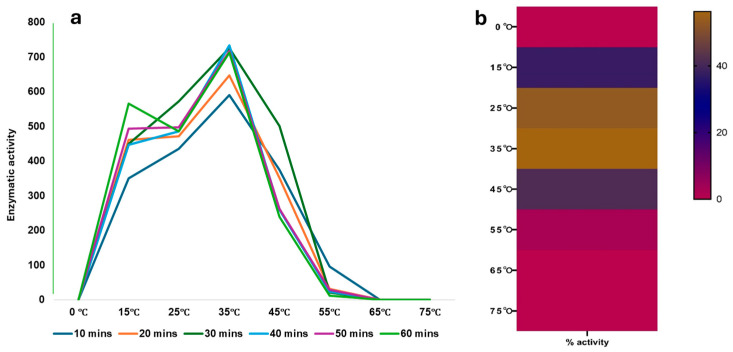
(**a**) Recombinant CmAP activity and stability at different temperatures. (**b**) Optimum temperature for enzymatic activity. Activity was measured by the hydrolysis of *p*NPP at 37 °C for 30 min, using a standard activity assay. The y-axis shows the relative enzymatic activity, determined by absorbance at OD_400_.

**Figure 5:**
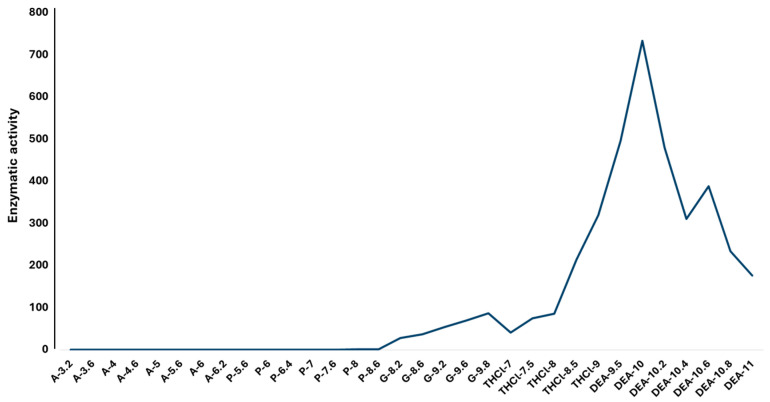
Recombinant CmAP activity under different buffers and pHs with *p*NPP as substrate: A—0.03 M acetate buffer (pH 3.2–6.2); P—phosphate buffer (pH 5.6–8.6); G—0.1 M glycine buffer (8.2–9.8); THCl—0.05 M Tris HCL (pH 7–9); DEA—1 M diethanolamine buffer (pH 9.5–11). The *y*-axis shows the relative enzymatic activity, determined by absorbance at OD_400_.

The recombinant enzyme rCmAP was exposed to different pHs and temperatures at varied time intervals to evaluate its stability. In general, rCmAP is relatively thermostable, retaining its activity when incubated at temperatures between 15 and 45 °C (up to 60 min) (Figure 4), with optimal activity at 35 °C and pH 10 using 0.1 M Tris-HCl or 1 M diethanolamine buffer (Figure 5).

The enzyme exhibited a significant reduction in activity at pH values above 10 and a complete loss of activity at temperatures above 55 °C (Figure 4 and Figure 5). Figure 4 and Figure 5 present detailed activity profiles under varying temperature and pH conditions, respectively.

### 2.4. Effect of Salt Concentration on rCmAP Activity

**Figure 6:**
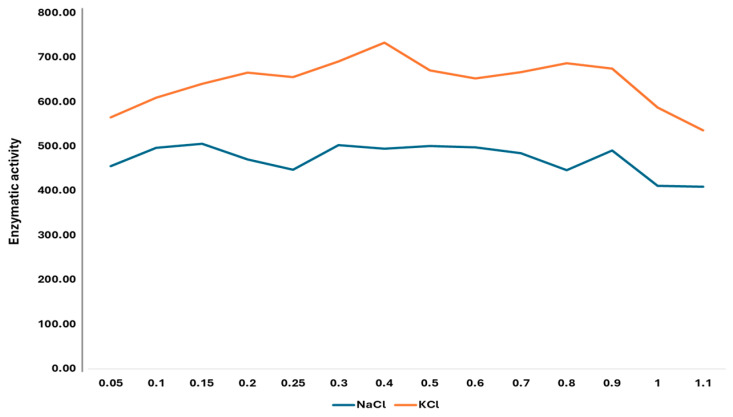
The effect of NaCl and KCl concentrations on rCmAP enzymatic activity was determined by the hydrolysis of *p*NPP at 37 °C over 30 min using the standard activity assay. The *y*-axis represents the relative enzymatic activity determined by absorbance at OD_400_.

The activity of rCmAP exhibited relative stability across a salt concentration range of 0.05–1.1 M, maintaining optimum enzymatic function (Figure 6).

A slight, though insignificant, decline in activity was observed at NaCl and KCl concentrations exceeding 1 M. Overall, rCmAP showed a higher enzymatic activity in KCl solutions than in NaCl at comparable concentrations (Figure 6).

### 2.5. Effect of Bivalent Metal Ions on rCmAP Activity

**Figure 7:**
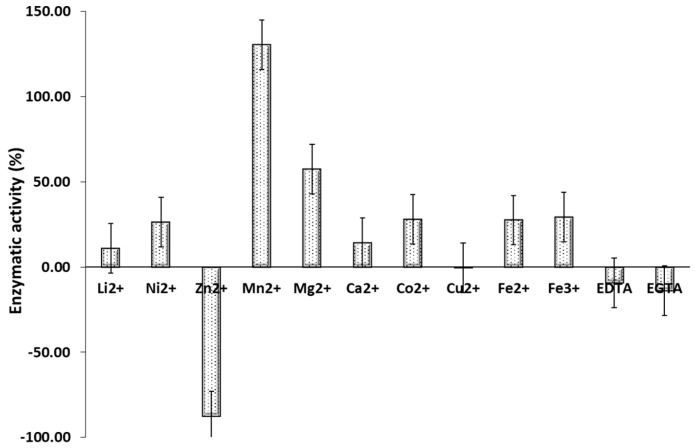
Effect of different bivalent and trivalent metal ions on rCmAP activity. The *y*-axis represents the relative rCmAP enzymatic activity (%) compared to the control reaction in the absence of bivalent metal salts, measured using the standard activity assay. Data are presented as mean ± standard error.

The results indicate that rCmAP activity depends on the presence of bivalent metal ions in the incubation medium (Figure 7).

In particular, rCmAP activity was significantly enhanced by manganese (Mn^2+^) and magnesium (Mg^2+^), with a marked increase of approximately 130% and 60%, respectively (Figure 7). In contrast, the enzyme’s activity was significantly inhibited by zinc (Zn^2+^) to near-zero levels, with a decrease of approximately 80%. Other bivalent metal ions, including lithium (Li^2+^), nickel (Ni^2+^), calcium (Ca^2+^), cobalt (Co^2+^), copper (Cu^2+^), and iron (Fe^2+^ and Fe^3+^), were found to vary, with no significant effect on the activity of the enzyme. The addition of EDTA and EGTA in rCmAP incubation medium, chelating agents, resulted in a decline in enzyme activity, suggesting the requirement of divalent metal ions for optimal enzyme function (Figure 7).

### 2.6. Screening rCmAP Preparation for LPS Contamination

**Figure 8:**
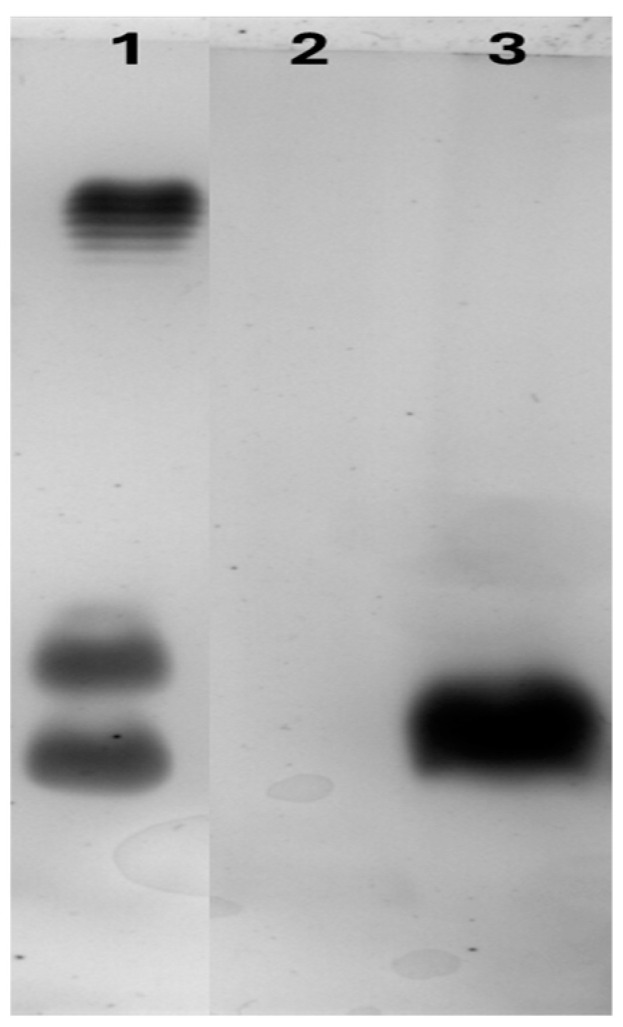
SDS-PAGE analysis for LPS contamination in recombinant CmAP from *N. tabacum* and *E. coli* expression systems, performed using 12% SDS-PAGE and silver staining. Lane 1: Control LPS derived from *E. coli* strain O55:B5 LPS (Sigma, St. Louis, MO, USA); Lane 2: Recombinant CmAP purified from tobacco leaves; Lane 3: Recombinant CmAP purified from *E. coli* [8].

Silver staining of SDS-PAGE gels revealed the presence of LPS molecules as clear dark bands in the *E. coli* LPS lysate and the *E. coli*-expressed rCmAP, indicating contamination of the expressed enzyme by the host bacterial LPS (Figure 8).

However, the protein solution of rCmAP purified from the tobacco leaves showed no evidence of LPS contamination, confirming its high-quality purity (Figure 8).

## 3. Discussion

Marine bacteria provide a vast repository of valuable enzymes with potential applications in industrial and therapeutic settings [4,24]. Alkaline phosphatases are among the most prevalent enzymes in soils and the ocean; they are indispensable for nutrient conversion as bacteria rely on them in inorganic phosphate (Pi)-deficient situations to solubilize different types of organic phosphates [25,26]. The marine bacterium *C. amphilecti* KMM 296, residing in the mussel gut, has garnered increasing scientific interest due to its highly active alkaline phosphatase (CmAP), belonging to the PhoA structural family. This enzyme has been proven highly effective in dephosphorylating *E. coli* LPS [4,11]. As a result, CmAP has become a focal point of research, as evidenced by numerous studies [4,6,7,8,11,16,27].

### 3.1. Differential Expression of rCmAP in Calli and Leaves

This study reports the successful expression of an active recombinant enzyme, rCmAP, in a plant system, thereby supporting earlier findings [28,29]. Notably, this is the first report of rCmAP production in plant systems. A total of 0.227 mg of rCmAP was obtained from 4.4 mg of the total protein in the homogenate of 2 g transiently transformed *N. tabacum* leaves (Figure 3, Table 1). The recombinant enzyme obtained exhibited high activity, with a specific enzymatic activity of 14.6 U/mg in tobacco leaves (Figure 3, Table 1), which is comparable to that of the enzyme produced by the native bacterium *C. amphilecti* KMM 296 (15 U/mg) [7] and slightly lower than that achieved in recombinant *E. coli* Rosetta DE (+) lysate (18.2 U/mg) [8]. Compared to tobacco leaves, the recombinant enzyme rCmAP exhibited very low expression levels and yields in the callus tissues despite their high total protein content (Figure 3). This observation is consistent with previous findings reporting higher recombinant protein expression in leaves than in callus and cell suspensions [30]. This disparity reflects the differential metabolic priorities of the tissues: callus tissues focus on active cell differentiation, growth, and expansion, thereby limiting resources for recombinant protein synthesis. In contrast, leaves exhibit greater metabolic stability, robust cellular machinery, and higher photosynthetic rates, providing the energy required for efficient protein synthesis and accumulation [31].

### 3.2. Expression Vectors Influence on rCmAP Yield

The two expression vectors, pHREAC-CmAP and pEff-CmAP, used in this study were designed specifically for high-yield recombinant protein production in transiently transformed tobacco leaves [22,23]. The impact is evident from the high recombinant protein yield and enzymatic activity detected in the transformed leaves (Table 1, Figure 3). The vector type had a negligible impact on rCmAP production when heterologously expressed in callus cultures (Figure 3). At the same time, a twofold increase in rCmAP expression was observed in *N. tabacum* leaves transfected with the pEff-CmAP-6H construct (14.6 U/mg) compared to the pHREAC-CmAP or pHREAC-CmAP-6H gene constructs (Figure 1 and Figure 3). The influence of vectors on recombinant gene expression in heterologous hosts cannot be overemphasized [32]. Although they shared the goal of optimizing protein expression, these vectors operate through distinct mechanisms. The pHREAC plasmid functions as an enhanced binary expression vector, featuring optimized untranslated regions (UTRs) that promote hyper-translation. It is non-replicating, relying solely on transcription and highly efficient translation to drive protein production. In contrast, the pEff vector incorporates a viral 5′-UTR and 3′-UTR for enhanced translation, as well as the RNA-dependent RNA polymerase (RDRP) from potato virus X (PVX), enabling mRNA replication. This replication feature will likely amplify transcript levels, thereby enhancing protein yield. Furthermore, pHREAC and pEff vectors contain silencing inhibitors, namely NSs and p24, respectively, to minimize post-transcriptional gene silencing, which often suppresses foreign gene expression. Our findings suggest that rCmAP production is more efficient when utilizing a replicating RNA viral vector, such as pEff, than a non-replicating pHREAC-like vector.

### 3.3. A Putative Influence of Post-Translational Modification on rCmAP Purification

The activity of rCmAP produced in plants and its reduced capacity to bind to Ni^2+^ and ion-exchange resins (Table 1) may be influenced by its glycosylation pattern. This indicates the potential for the recombinant enzyme to undergo post-translational modification by the host plant’s protein modification machinery. It is primarily the function of plants to add complex *N*-glycans that terminate in mannose or N-acetylglucosamine [33]. It is also noteworthy that D-xylose is β-1,2-linked to the core β-linked mannose, and L-fucose is α-1,3-linked to the first core *N*-acetylglucosamine (GlcNAc) residue. This glycosylation pattern differs significantly from the human type, potentially posing a challenge for plant-based production of human proteins due to the plant-specific glycoforms, which have the potential to elicit immunogenic responses against recombinant proteins [34,35]. In contrast to eukaryotic proteins, bacterial proteins do not undergo such post-translational modifications that are unnecessary for their function and could potentially impair their enzymatic activity [36,37]. An analysis of the CmAP amino acid sequence using GlycoPP V 1.0 [38] and NetNGlyc-1.0 [39] revealed the *N*-linked glycosylation site in the CmAP protein. The occurrence of the ‘Asparagine-X-Threonine’ (NGT) tripeptide sequence at the 212th position of the CmAP peptide chain, which is recognizable by plant cellular machinery, has a high probability of undergoing *N*-glycosylation. This could have signaled the eukaryotic host system to attach an *N*-linked glycan to the asparagine residue. A study by Mamedov et al. [37] using *N. benthamiana* revealed that bacterial proteins lacking *N*-linked glycans in their native hosts exhibit reduced biological activity when expressed in plants. This is due to the aberrant *N*-glycosylation encountered in the host plant. In a recent study, Kao et al. [40] observed that plant-glycosylated proteins may exhibit altered conformations or improper interactions with target molecules, reinforcing earlier claims [41]. However, further investigation is required to determine whether the rCmAP site is glycosylated in tobacco cells and, if so, how this may affect the rCmAP’s biological activity, affinity to Ni-Sepharose and MonoQ adsorbent, and digestibility by enterokinase (Table 1). Nevertheless, the 6xHis-tag of the plant-derived rCmAP exhibits a reduced affinity for traditional adsorbents compared to the bacterial recombinant CmAP [8].

### 3.4. Recombinant CmAP Characterization

The rCmAP protein was identified as a single band on SDS-PAGE with a molecular mass of approximately 55 kDa, consistent with previous findings for the native and bacterially expressed forms of the protein [4,7,8]. The rCmAP purified from tobacco leaves demonstrated remarkable thermostability and mesophilic properties, comparable to earlier reports on the native enzyme [7] and rCmAP expressed in *E. coli* [8]. The enzyme retained its activity when incubated for up to 60 min at temperatures ranging from 15 to 45 °C (Figure 4). The rCmAP exhibited an optimum temperature of 35 °C, slightly lower than the 40–50 °C range previously reported for the enzyme expressed in *E. coli* [6,8]. At temperatures above 55 °C, rCmAP becomes inactive and unstable, whereas the native enzyme retains activity up to 60 °C [7].

The optimal pH for rCmAP activity in this study was within the range of 10 to 10.5 when tested in 1 M diethanolamine (DEA) buffer or 0.1 M Tris-HCl. Beyond this pH range, the rCmAP activity dropped sharply (Figure 5). The optimal pH value is consistent with the native enzyme [7] and is slightly higher than the pH range of 9.5–10.3 reported for the recombinant CmAP produced in *E. coli* in 1 M DEA buffer [8]. It is hypothesized that during the expression process in tobacco, subtle structural modifications to rCmAP may occur, potentially altering its interaction with water molecules and influencing its pKa values [40].

The findings of this study indicate that rCmAP is relatively stable across a range of KCl concentrations, following the observations of Golotin et al. [8]. However, the stability of rCmAP in NaCl differs from the reported findings, which indicated a decline in enzymatic activity at NaCl concentrations above 0.25 M [8]. As noted in earlier studies, the rCmAP activity was consistently higher in KCl compared to NaCl at equivalent concentrations [7,8]. Despite fluctuations in activity across the tested salt concentrations (0.05–1.1 M), these variations were largely insignificant (Figure 6). The rCmAP activity declined at salt concentrations above 1 M (Figure 6). The recombinant enzyme demonstrated greater salt tolerance compared to the native enzyme, whose activity steadily decreased at NaCl concentrations above 0.2 M [7,8]. The recombinant enzyme’s increased salt tolerance renders it a suitable candidate for several biotechnological applications, including bioremediation in saline environments [42] and the food industry, particularly in producing fermented foods under high salt concentrations and, similarly, in pharmaceuticals, where salt is frequently employed in drug formulation and purification to stabilize proteins and other biomolecules [43]. Additionally, the results confirmed that the rCmAP activity is bivalent metal-dependent. The presence of Mn^2^⁺ and Mg^2^⁺ enhanced its activity, while EDTA and Cu^2^⁺ had a minimal impact. In contrast, Zn^2^⁺ drastically reduced CmAP activity to near-zero levels, a finding consistent with the behavior of the native and recombinant CmAP enzymes [7,8].

### 3.5. Tobacco Expression System Yields LPS-Free Recombinant CmAP

Recombinant CmAP obtained from *N. tabacum* is free from lipopolysaccharide (LPS) contamination, unlike the recombinant CmAP expressed in *E. coli* systems (Figure 8). While *E. coli* offers a well-optimized protein expression system, its transcription, translation, and protein folding machinery are adapted for high-level protein production [44,45]. This frequently results in the accumulation of recombinant CmAP within the bacterial cytoplasm, as demonstrated in previous studies [4,6,7,8]. In addition, the recombinant CmAP derived from *E. coli* systems is inevitably contaminated with LPS, a natural component of the outer membrane of Gram-negative bacteria. LPS contains the highly bioactive lipid ‘A’ moiety, which can trigger severe endotoxin responses even in trace amounts [46].

The presence of LPS contamination in rCmAP produced in *E. coli* without the additional step of purification for LPS removal was confirmed (Figure 8). This contamination renders the enzyme unsuitable for pharmacological applications, as it could induce strong immune responses in humans, including fever, chills, inflammation, and, in severe cases, septic shock [46,47]. Additionally, although *E. coli* strains used for recombinant protein production are non-pathogenic, there is a potential risk of horizontal gene transfer, leading to the accidental acquisition of virulence genes or antibiotic resistance elements from contaminating *E. coli* strains in the culture [48]. These limitations further restrict its use in human applications.

In contrast, plant-based expression systems, such as *Nicotiana* spp., are naturally free from LPS and other microbial contaminants [49,50]. While researchers have developed several protein purification procedures to remove LPS contaminants from recombinant enzymes, plant expression systems eliminate the need for these additional procedures, which demand additional time and resources. This study successfully establishes the expression of CmAP in tobacco plants as a safe and scalable alternative for therapeutic and industrial applications. Future efforts should focus on optimizing the enzymatic yield and activity for enhanced production.

## 4. Materials and Methods

### 4.1. Gene Cloning and Vector Construction

The plasmids pHREAC [22] and pEff [23], which facilitate efficient transient overexpression of recombinant proteins in plants, were obtained from Addgene (https://www.addgene.org/ accessed on 4 March 2024). The *C. amphilecti alp* gene without its signal peptide (corresponding to positions 30,149–31,660 bp in GenBank Acc. No. JQJA01000018) was PCR-amplified from a pET-40b(+) vector encoding *C. amphilecti* KMM 296 mature alkaline phosphatase retrieved from previous research [8]. The CDS, as well as a 3D structure of mature CmAP, is presented as Supplementary Materials. Amplification was performed in a 20 µL polymerase chain reaction (PCR) volume with Encyclo polymerase (Evrogen, Moscow, Russia) using a Bio-Rad thermocycler C1000 Touch^TM^ (Bio-Rad, CA, USA). The PCR products were visualized using gel electrophoresis (1.2% Agarose gel in 1X TAE Buffer); the *C. amphilecti alp* gene was confirmed as a 1500 Kb band as earlier described for mature CmAP [8].

Three expression vectors were constructed to enable *C. amphilecti alp* expression under different configurations. Gene expression was driven by the 35S CaMV promoter in the plasmids pHREAC and pEff. The codon structure of the gene was not optimized for the heterologous system, as native expression levels were sufficient for our objectives. The gene structure comprises a simple CDS without introns. First, the *alp* gene was amplified using primers CmAP-D (5′-TAT GGT CTC AAA AAA TGG CAG AGA TCA AGA ATG TCA TTC-3′) and CmAP-R (5′-TAT GGT CTC TAG CGC TAG CGT CAA TGG TGA TGG TGA TGG TGC TTC GCT ACC ACT GTC TTC-3′) and cloned into the pHREAC vector through *Bsa*I sites, yielding the pHREAC-CmAP construct without any additional modifications. To create a variant with a 6xHis tag at the C-terminus, separated by an enterokinase site, the *C. amphilecti alp* gene was amplified with primers CmAP-D and CmAP-R2 (5′-TAT GGT CTC TAG CGC TAG CGT CAA TGG TGA TGG TGA TGG TGC TTG TCG TCG TCA TCC TTC GCT ACC ACT GTC TTC-3′). This amplified product was also inserted into pHREAC via *Bsa*I sites, producing the pHREAC-CmAP-6H construct. Finally, to express the *alp* gene in the pEff vector, we used the primers pEff-CmAP-D (5′-TAT GGC GCG CCA TGG CAG AGA TCA AGA ATG TCA TT-3′) and pEff-CmAP-R (5′-ATA CCC GGG TCA ATG GTG ATG GTG ATG GTG CTT GTC GTC GTC ATC CTT CGC TAC CAC TGT CTT-3′) to amplify the gene with a 6xHis tag and an enterokinase site at the C-terminus. This product was inserted into pEff using *Asc*I and *Sma*I restriction sites, yielding the final pEff-CmAP-6H construct. Each construct was verified through sequencing with primers specific to the pHREAC and pEff backbone, located upstream and downstream of the *C. amphilecti alp* gene insert, using an ABI 3500 Genetic Analyzer (Applied Biosystems, Foster City, CA, USA) as described in prior studies [51].

### 4.2. Preparation of Agrobacterium tumefaciens Strains

The final constructs (pHREAC-CmAP, pHREAC-CmAP-6H, and pEff-CmAP-6H) were introduced into *Agrobacterium tumefaciens* strain EHA105/pTiBo542 [52] by electroporation using a Gene Pulser (Bio-Rad Laboratories, Inc., Hercules, CA, USA) following the manufacturer’s protocol. Transformed cells were plated on Luria–Bertani (LB) agar containing 50 mg/L kanamycin and 50 mg/L rifampicin and incubated at 28 °C for 48 h. Positive colonies were confirmed by PCR with gene-specific primers and transferred to liquid LB media containing kanamycin and rifampicin, where they were grown to an OD_600_ of 1.0. The bacterial culture was pelleted by centrifugation at 3000× *g* for 10 min and resuspended in 100 mL infiltration buffer (10 mM MES, pH 5.6; 10 mM MgCl_2_; 200 µM acetosyringone) to reach a final OD_600_ of 0.1. This suspension was incubated at room temperature for 2 h before infiltration.

### 4.3. Nicotiana tabacum Transient Transformation and Callus Induction

Using a needleless syringe, *A. tumefaciens* EHA105 clones carrying vectors with the target gene were infiltrated into the leaves of 4-week-old *N. tabacum* plants using infiltration buffer prepared as previously described [53]. To induce callus growth, the leaf-disk method was adopted following established protocols [54,55] with some modifications. Leaf discs: 3 mm^2^ sections were made across the leaf area of young, healthy leaves of *N. tabacum*, grown in vivo, intersecting the leaf veins. Individual *A. tumefaciens* EHA105 clones, each containing different expression cassettes, were used to inoculate the leaf discs under aseptic conditions and then cultured on Murashige and Skoog (MS) medium [56] supplemented with 0.5 mg/L 2,4-D, 2 mg/L IAA, and 0.5 mg/L BAP. To establish stable transgenic cultures, all transformed callus lines were subjected to 3–4 selection rounds on media containing 50 mg/L kanamycin. Following the selection process and confirmation of transgenicity, the cultures were transferred to an antibiotic-free medium and maintained for two passages before proceeding with further experiments. Calli were grown at 24 °C under a 24 h-dark regimen.

### 4.4. Recombinant Protein Purification

Six days post-infiltration, the transiently transformed leaves were harvested, flash-frozen in liquid nitrogen, ground into a fine powder using a pestle and mortar, and stored at −80 °C until further extraction. Similarly, callus biomass was collected from three-week-old actively growing cultures and subjected to the same treatment. The powdered mass was weighed and resuspended in extraction buffer (50 mM Tris HCl, pH 8.0) at 2:10 (*w*/*v*). The suspension was homogenized using an ultrasonic homogenizer (Bandelin Sonoplus UW 2070, Berlin, Germany) for 15 min (pulse on/off time of 40 s/20 s) in an ice bath, filtered, and centrifuged at 11,000× *g* for 20 min at 4 °C. The resulting supernatant was collected as a crude protein extract.

#### 4.4.1. Nickel-Sepharose Affinity Chromatography

The crude protein extract was loaded onto a 25 × 3.2 cm nickel-sepharose column (Cytiva (GE Healthcare) Life Sciences, Buckinghamshire, UK), which had been pre-equilibrated with binding buffer A (50 mM Tris-HCl, pH 8.0), and subsequently washed with five column volumes of the same buffer. The recombinant protein was eluted with a linear gradient of 0–0.5 M imidazole in 50 mM Tris-HCl buffer, pH 8.0, and 0.5 M NaCl (six-column volume) at a 1 mL/min flow rate.

#### 4.4.2. MonoQ Anion Exchange Chromatography

The fraction containing CmAP was loaded onto a pre-equilibrated 10 × 1.4 cm of MonoQ (Cytiva (GE Healthcare) Life Sciences) balanced with buffer A with 2 mM MgCl_2_ (buffer B). CmAP was eluted with a linear gradient of NaCl (0–0.5 M) in buffer B at a rate of 1 mL/min; fraction volume 1 mL.

### 4.5. Protein Quantification and Enzyme Activity Assay

The Bradford assay [57] was used with bovine serum albumin as a standard to determine the recombinant protein concentration in each fraction. Enzymatic activity was measured by the hydrolysis of 15 mM *p*-nitrophenyl phosphate (*p*NPP) under buffer (0.1 M Tris HCl (pH 10.0), 0.2 M KCl) for 30 min at 37 °C. The reaction was stopped by adding 2 mL of 0.5 M NaOH, and absorbance was measured at 400 nm using a spectrophotometer (Cecil CE 1021 UV/VIS, London, UK). One unit of enzyme activity was taken as the quantity of enzyme needed to hydrolyze 1 µmol of *p*NPP per minute.

### 4.6. Enzymatic Temperature and Optimum Temperature

To determine the optimal temperature for the enzyme activity, a standard incubation mixture should be used at temperatures from 10 to 75 °C at 10-min intervals, up to 1 h. The enzyme was cooled, and the residual activity was assessed using the standard activity assay.

### 4.7. Effects of Salts, Buffers, and pH on CmAP Activity

CmAP activity was assessed under varying concentrations of NaCl and KCl (0.05–1.6 M) and different buffers and pHs (0.03 M acetate buffer (pH 3–6.2); phosphate buffer (pH 5.6–8.6); 0.1 M glycine buffer (8.2–9.8); 0.05 M Tris HCL (pH 7–9); and 1 M diethanolamine buffer (pH 9.2–11). Standard enzyme activity assay was prepared as earlier described, using experimental buffers. After incubation, absorbance at A_400_ was recorded, and specific activity was estimated.

### 4.8. Effect of Bivalent Metal Ions on CmAP Activity

The standard enzyme activity assay was supplemented with 2 mM of different bivalent metals in their chloride/sulfate (LiCl_2_, NiCl_2_, ZnSO_4_, MnCl_2_, MgCl_2_, CaCl_2_, CoCl_2_, FeSO_4_, FeCl_3_), EDTA, and EGTA against the control enzyme activity assay without metal ions and chelate reagents to probe CmAP dependence on their presence. Specific activity was estimated as earlier described.

### 4.9. Screening CmAP for Glycosylation Sites

Glycosylation sites in CmAP were predicted using GlycoPP V 1.0 [38] and NetNGlyc-1.0 [39]. The mature CmAP amino acid sequence was submitted and analyzed on both servers.

### 4.10. Screening Recombinant Protein for LPS Contamination

Sodium Dodecyl Sulfate Polyacrylamide Gel Electrophoresis (SDS PAGE) was conducted to screen the purified recombinant protein for LPS contamination in comparison with *E. coli*-expressed CmAP and crude *E. coli* LPS as reference (obtained from G.B. Elyakov Pacific Institute of Bioorganic Chemistry, Far Eastern Branch, Russian Academy of Sciences) [8]. Protein samples were resolved on a 12% PAGE following the Lammley protocol [58]. To visualize LPS, the plate was fixed in 40% ethanol and 5% NaOH overnight, then oxidized in 0.7% HIO_4_, 40% ethanol, and 5% AcOH for 5 min and stained with Argentum using a freshly prepared solution containing 28% NH_4_OH, 0.1 N NaOH, 20% AgNO_3_, and dH_2_O, then thoroughly washed with dH_2_O. Gels were developed in a solution of 5% citric acid and 37% formaldehyde, with LPS appearing as distinct dark bands.

### 4.11. Statistical Analysis

Statistical tests were performed using Statistica 10.0 (StatSoft Inc., Tulsa, OK, USA), with the threshold for statistical significance set at *p* < 0.05.

## 5. Conclusions

This research successfully establishes the production of LPS-free *C. amphilecti* alkaline phosphatase CmAP in *N. tabacum* leaves and calli using *Agrobacterium*-mediated transformation methods. The transient transformation of tobacco leaves was found to be an effective method for producing the marine bacterial recombinant enzyme rCmAP, which was functional and active and exhibited thermostability, salt tolerance, and other properties comparable to those of the native enzyme.

However, the production of rCmAP in stably transformed callus cultures was notably weak, with low expression levels and yield despite high total protein values in the tissues. This difference in production efficiency between callus and leaves highlights the metabolic distinctions between these tissues, with callus prioritizing active cell differentiation and growth over recombinant protein synthesis.

The study also emphasizes the significant influence of expression vectors on CmAP yield and activity. The replicating RNA viral vector ‘pEff’ yielded a twofold higher level of the enzymatically active rCmAP compared to the non-replicating vector. This work lays the foundation for the production of LPS-free CmAP in tobacco plants, demonstrating its potential as a scalable platform. However, further research is required to optimize the yield and activity of the bacterial recombinant PhoA family ALP, particularly in callus cultures, to support therapeutic applications.

## Figures and Tables

**Table 1:** Purification yields and specific activities for rCmAP expressed in *N. tabacum* leaves * and *E. coli*.

*N. tabacum*/*E. coli*	Total Protein (mg)		Specific Activity (U/mg)		Total Activity (U)	
Homogenate	4.40 ± 0.29	378.00 ± 8.34	14.60 ± 0.88	101.60 ± 5.10	64.24 ± 3.48	38,404.80 ± 212.30
Ni-Sepharose elute	1.06 ± 0.20	108.40 ± 4.45	113.20 ± 2.89	787.50 ± 12.23	119.90 ± 4.41	85,365.00 ± 182.65
Dialysis	1.15 ± 0.15	11.50 ± 1.20	136.84 ± 5.77	853.20 ± 9.10	157.09 ± 4.62	9811.80 ± 75.20
HisTrap	0.69 ± 0.12	3.10 ± 0.23	318.84 ± 8.82	1984.60 ± 21.12	219.36 ± 5.01	6152.30 ± 89.08
Mono-Q elute	0.23 ± 0.04	1.40 ± 0.12	733.30 ± 15.28	4052.60 ± 32.87	166.50 ± 5.77	5673.60 ± 76.90

* For rCmAP purification, 2 g of *N. tabacum* leaves were homogenized in 50 mM Tris-HCl buffer, pH 8.0. Data are presented as mean ± standard error.

## Data Availability

The original contributions presented in the study are included in the article; further inquiries can be directed to the corresponding author.

## Supplementary Materials

The following supporting information can be downloaded at: https://www.mdpi.com/article/10.3390/plants13243570/s1, CDS and amino acid sequence of matured CmAP, CDS and amino acid sequence of rCmAP, Figure S1: Homology model of matured CmAP generated using Swiss-model.
