# Supplementary material for: Expression and Characterization of Alkaline Phosphatase from Cobetia amphilecti KMM 296 in Transiently Transformed Tobacco Leaves and Transgenic Calli
Source: Plants (Basel). 2024 Dec 21;13(24):3570. doi: 10.3390/plants13243570 (PMC11679904; doi:10.3390/plants13243570)
Supplement: Supplementary file 1 [file plants-13-03570-s001.zip › plants-3338302-supplementary.pdf]

Supplementary data

CDS of matured *Cobetia amphilecti* alkaline phosphatase (CmAP)

ATGGCAGAGATCAAGAATGTCATTCTGATGATTGGCGATGGCATGGGGCCCCAGCAGGTTG
GCATGCTGGAGACATACGCCAATCGTGCGCCGGATTCGATATATCAAGGGCGTTCGACGGC
ACTCTACCAGCTGGCGAAGGAAGGCGTGGTGGGCGCTTCATTGACTCACCCTGAAGATGCCG
TGGTAGTGGATTCGGCCTGTTCAGCGACTCAGCTCTCGACGGGTATCTTCACCGGGGGCGAG
GTGATCGGCATAGATTCAGAGGGTAATCGGGTGGAAACCGTCCTCGAGCTTGCCAAACGAG
TCGGCAAGGCCACCGGATTGGTGTCCGATACGCGCCTGACCCACGCGACTCCGGCGGCCTTC
GCCGCCCACCAGCCGCATCGTTCACTGGAAAATGCCATTGCCGAAGACATGCTCATGACTGG
GCCGGATGTCATGCTGTCGGGAGGGTTGCGTCACTTCGTGCCATACTCCGTCAGTGAGCCGG
GGGAAAGCGCGGGCAGTGTCGAGACGCTGATGCAAGGTGCCTGGTCGCCAACCTCCAAGCG
CAAGGATGAACGAAATCTGCTCCAGGAAGCTGCAGATCAAGGCTATGGGCTAGCCTTTACG
CGTGATCAGATGGCCGCACTCAATGGCACCAAGGTATTGGGGCTATTCGCCAACTCGGGTAT
GGCGGATGGTATCAGCTTTCGAGATAGCCATGATGACCCACAGCGTCAGCAGCCGACCCTGC
ATGAGATGACCCAAAAAGCACTGTCAATGCTTGAACAGGATGATGATGGTTTCTTTTTGATG
GTCGAGGGTGGCCAGATCGACTGGGCCGCGCATTCCAATGATGCGGGCACCATGCTCAATG
AACTGATCAAGTTCGATGAGGCCGTGCAAGGTGTCTTTGACTGGGCGCGAGACCGAGACGA
TACAATAATCCTGGTAACGGCCGACCATGAGACGGGAGCATTCGGCTTCAGTTATTCCAGCG
CCAATTTACCGGCAGCGCAGAAGAAATCAGGCCCTGCATTCGCAGATCAGGATTACGCTCCC
AATTTCAATTTTGGTGACTTCTCGATTCTGGACAGTCTCTATGAGCAGAAGCAGACCTATTAC
GAGCTGCTCAGTGATTTCGAGGCACTACCGCAAGGTGAGCGCACACCGGCTCGCTTGATGGC
GGCTGTCAATGGCAATAGTGACTTCCAGATTACCGAAGCCCAGGCAGCTGAAGTACTGGCC
AACAAGCCCAACCCCTATCATGTGGATGGACATAGTTACCTCGGGGTCAGCGAGGTGCCGG
CAGTTCACGACTTCGACGCCTTCTTCCCCTATAACGATCGCGGCAACCTGCTGGCGCGGGCA
TTGGCGACACAGCAAAACACCGTCTGGGGGACTGGTACGCACACCCATACGCCAGTCAACG
TCTTTGCATGGGGGCCAGCCAACGACATCTTGCCGGTCTCTTCCATCCTGCACCATTCCGAGA
TAGGACAGTATCTGAAGACAGTGGTAGCGAAGTAA

Amino acid sequence of matured CmAP

AEIKNVILMIGDGMGPQQVGMLETYANRAPDSIYQGRSTALYQLAKEGVVGASLTHPE
DAVVVDSACSATQLSTGIFTGGEVIGIDSEGNRVETVLELAKRVGKATGLVSDTRLTHA
TPAAFAAHQPHRSLENAIAEDMLMTGPDVMLSGGLRHFVPYSVSEPGESAGSVETLMQ
GAWSPTSKRKDERNLLQEAADQGYGLAFTRDQMAALNGTKVLGLFANSGMADGISFR
DSHDDPQRQQPTLHEMTQKALSMLEQDDDGFFLMVEGGQIDWAAHSNDAGTMLNELI
KFDEAVQGVFDWARDRDDTIILVTADHETGAFGFSYSSANLPAAQKKSGPAFADQDYA
PNFNFGDFSILDSLYEQKQTYYELLSDFEALPQGERTPARLMAAVNGNSDFQITEAQAAE
VLANKPNPYHVDGHSYLGVSEVPAVHDFDAFFPYNDRGNLLARALATQQNTVWGTGT
HTHTPVNVFAWGPANDILPVSSILHHSEIGQYLKTVVAK

CDS of recombinant *Cobetia amphilecti* alkaline phosphatase (rCmAP)

ATGGCAGAGATCAAGAATGTCATTCTGATGATTGGCGATGGCATGGGGCCCCAGCAGGTTG
GCATGCTGGAGACATACGCCAATCGTGCGCCGGATTCGATATATCAAGGGCGTTCGACGGC
ACTCTACCAGCTGGCGAAGGAAGGCGTGGTGGGCGCTTCATTGACTCACCCTGAAGATGCCG
TGGTAGTGGATTCGGCCTGTTCAGCGACTCAGCTCTCGACGGGTATCTTCACCGGGGGCGAG
GTGATCGGCATAGATTCAGAGGGTAATCGGGTGGAAACCGTCCTCGAGCTTGCCAAACGAG
TCGGCAAGGCCACCGGATTGGTGTCCGATACGCGCCTGACCCACGCGACTCCGGCGGCCTTC
GCCGCCCACCAGCCGCATCGTTCACTGGAAAATGCCATTGCCGAAGACATGCTCATGACTGG
GCCGGATGTCATGCTGTCGGGAGGGTTGCGTCACTTCGTGCCACAGTCCGTCAGTGAGCCGG
GGGAAAGCGCGGGCAGTGTCGAGACGCTGATGCAAGGTGCCTGGTCGCCAACCTCCAAGCG

CAAGGATGAACGAAATCTGCTCCAGGAAGCTGCAGATCAAGGCTATGGGCTAGCCTTTACG
CGTGATCAGATGGCCGCACTCAATGGCACCAAGGTATTGGGGCTATTCGCCAACTCGGGTAT
GGCGGATGGTATCAGCTTTCGAGATAGCCATGATGACCCACAGCGTCAGCAGCCGACCCTGC
ATGAGATGACCCAGAAAGCACTGTCAATGCTTGAACAGGATGATGATGGTTTCTTTTTGATG
GTCGAGGGTGGCCAGATCGACTGGGCCGCGCATTCCAATGATGCGGGCACCATGCTCAATG
AACTGATCAAGTTCGATGAGGCCGTGCAAGGTGTCTTTGACTGGGCGCGAGAGCGAGACGA
TACAATAATCCTGGTAACGGCCGACCATGAGACGGGAGCATTCGGCTTCAGTTATTCCAGCG
CCAATTTACCGGCAGCGCAGAAGAAATCAGGCCCTGCATTCGCAGATCAGGATTACGCTCCC
AATTTCAATTTTGGTGACTTCTCGATTCTGGACAGTCTCTATGAGCAGAAGCAGACCTATTAC
GAGCTGCTCAGTGATTTCGAGGCACTACCGCAAGGTGAGCGCACACCGGCTCGCTTGATGGC
GGCTGTCAATGGCAATAGTGACTTCCAGATTACCGAAGCCCAGGCAGCTGAAGTACTGGCC
AACAAGCCCAACCCCTATCATGTGGATGGACATAGTTACCTCGGGGTCAGCGAGGTGCCGG
CAGTTCACGACTTCGACGCCTTCTTCCCCTATAACGATCGCGGCAACCTGCTGGCGCGGGCA
TTGGCGACACAGCAAAACACCGTCTGGGGGACTGGTACGCACACCCATACGCCAGTCAACG
TCTTTGCATGGGGGCCAGCCAACGACATCTTGCCGGTCTCTTCCATCCTGCACCATTCCGAGA
TAGGACAGTATCTGAAGACAGTGGTAGCGAAGGATGACGACGACAAGCACCATCACCATCA
CCATTGA

Amino acid sequence of recombinant CmAP
AEIKNVILMIGDGMGPQQVGMLETYANRAPDSIYQGRSTALYQLAKEGVVGASLTHPE
DAVVVDSACSATQLSTGIFTGGEVIGIDSEGNRVETVLELAKRVGKATGLVSDTRLTHA
TPAAFAAHQPHRSLENAIAEDMLMTGPDVMLSGGLRHFVPQSVSEPGESAGSVETLMQ
GAWSPTSKRKDERNLLQEAADQGYGLAFTRDQMAALNGTKVLGLFANSGMADGISFR
DSHDDPQRQQPTLHEMTQKALSMLEQDDDGFFLMVEGGQIDWAAHSNDAGTMLNELI
KFDEAVQGVFDWARERDDTIILVTADHETGAFGFSYSSANLPAAQKKSGPAFADQDYA
PNFNFGDFSILDSLYEQKQTYYELLSDFEALPQGERTPARLMAAVNGNSDFQITEAQAAE
VLANKPNPYHVDGHSYLGVSEVPAVHDFDAFFPYNDRGNLLARALATQQNTVWGTGT
HTHTPVNVFAWGPANDILPVSSILHHSEIGQYLKTVVAK*DDDDK***HHHHHH**

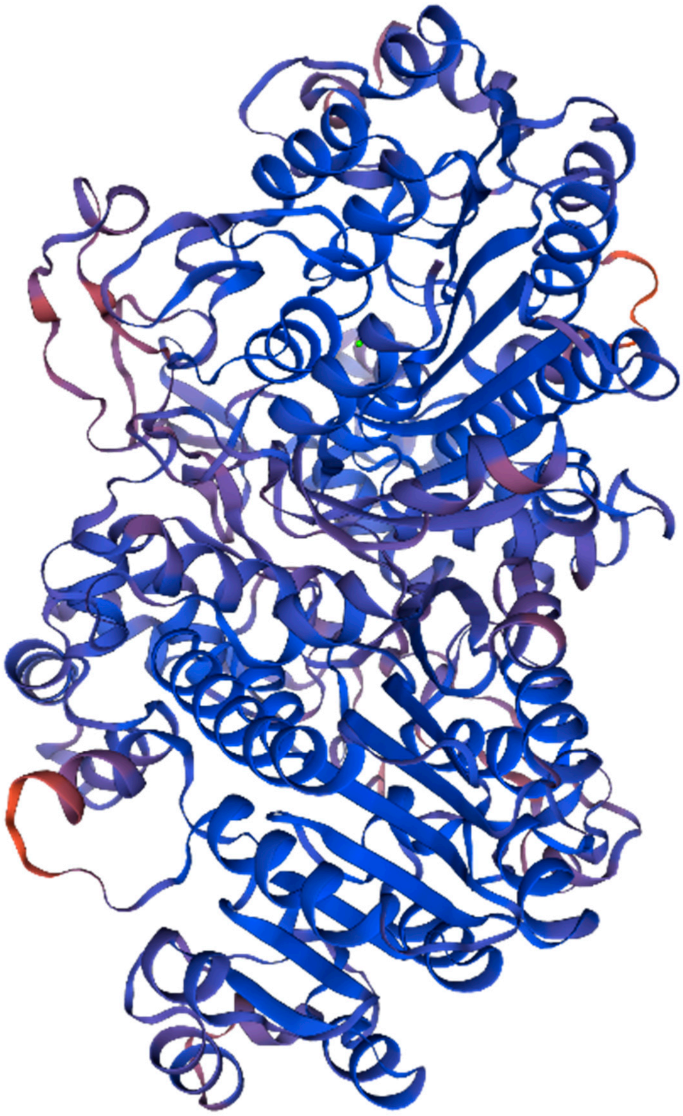

Supplementary Figure 1: Homology model of matured CmAP generated using Swiss-model (https://swissmodel.expasy.org/)
